# Supplementary material for: Rootlets-based registration to the PAM50 spinal cord template
Source: Imaging Neurosci (Camb). 2025 Aug 26;3:IMAG.a.123. doi: 10.1162/IMAG.a.123 (PMC12381661; doi:10.1162/IMAG.a.123)
Supplement: Supplementary Material [file IMAG.a.123_supp.pdf]

13. Supplementary Material

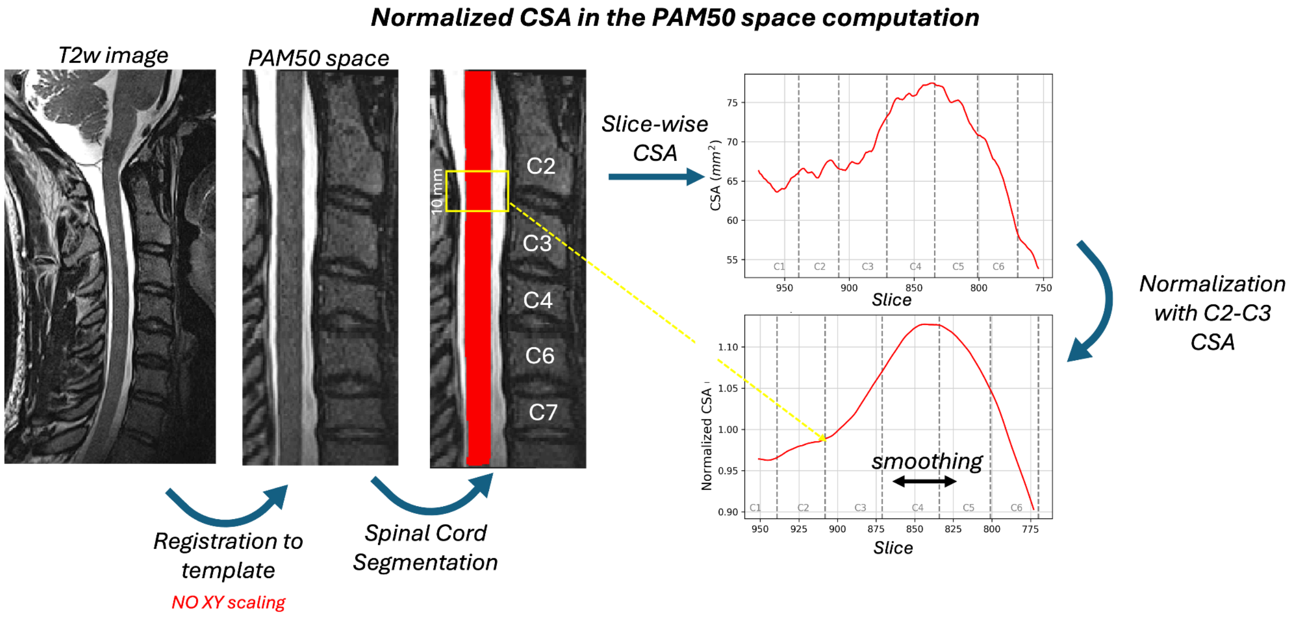

Figure S1. ***Pipeline to analyse spinal cord morphometry.*** *First, the T2w image is registered to the PAM50 template using either rootlet-based or disc-based registration without the axial scaling of the spinal cord shape (xy-plane) to avoid changing the spinal cord morphology. Then, the spinal cord was segmented and the spinal cord cross-sectional area (CSA) was computed for each slice. CSA was normalized to the over 20 slices (10 mm) centered at C2–C3 intervertebral disc level and smoothed using a 45-slice moving average filter window.*

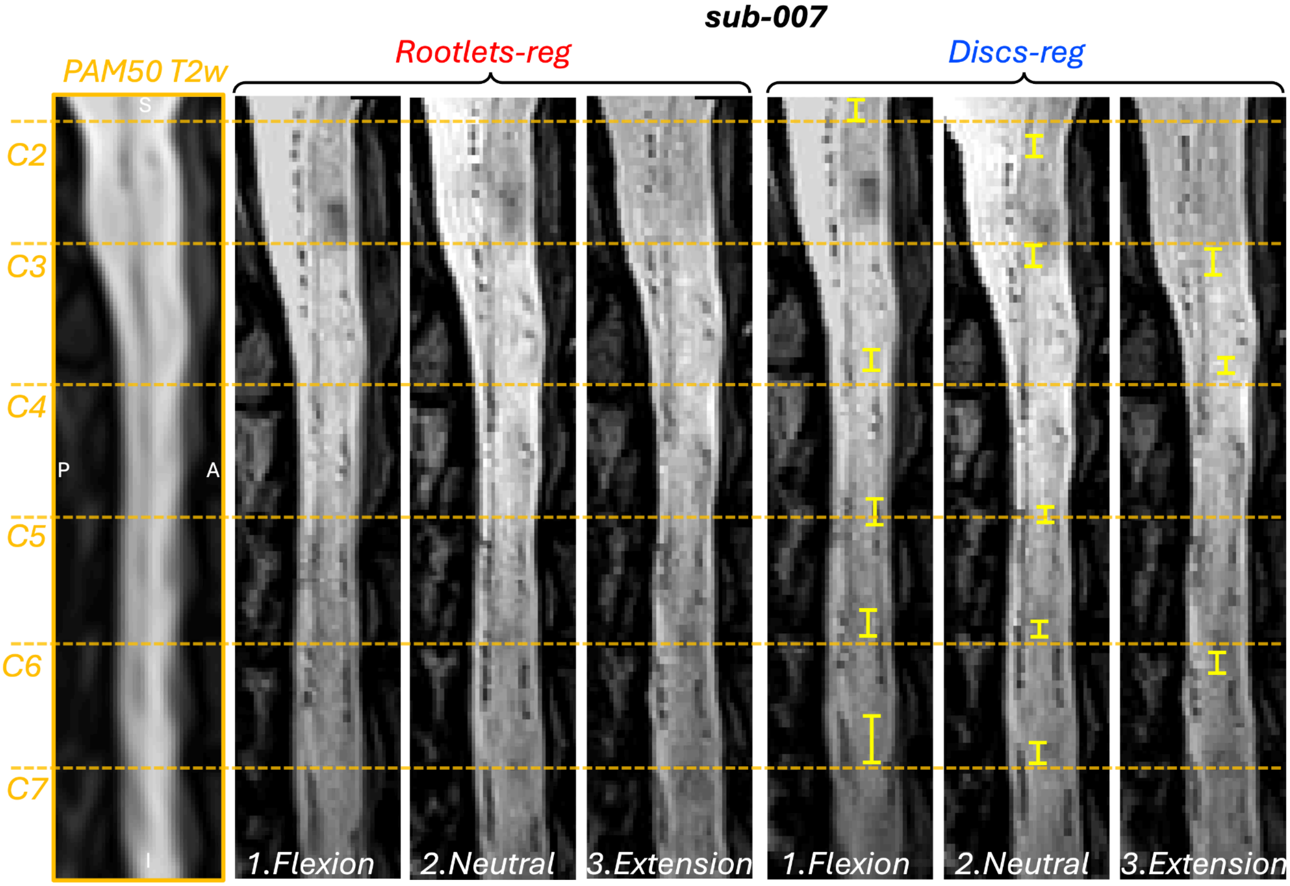

Figure S2. *Registration results for participant sub-007, showing spinal cord alignment using rootlet-based (red) and disc-based (blue) registration in all three neck positions. The PAM50 sagittal slice (x=84) is displayed for reference. The orange dashed lines mark the top of spinal levels C2-C7. Yellow brackets highlight misalignment of dorsal rootlets in the disc-based registration method compared to the PAM50.*

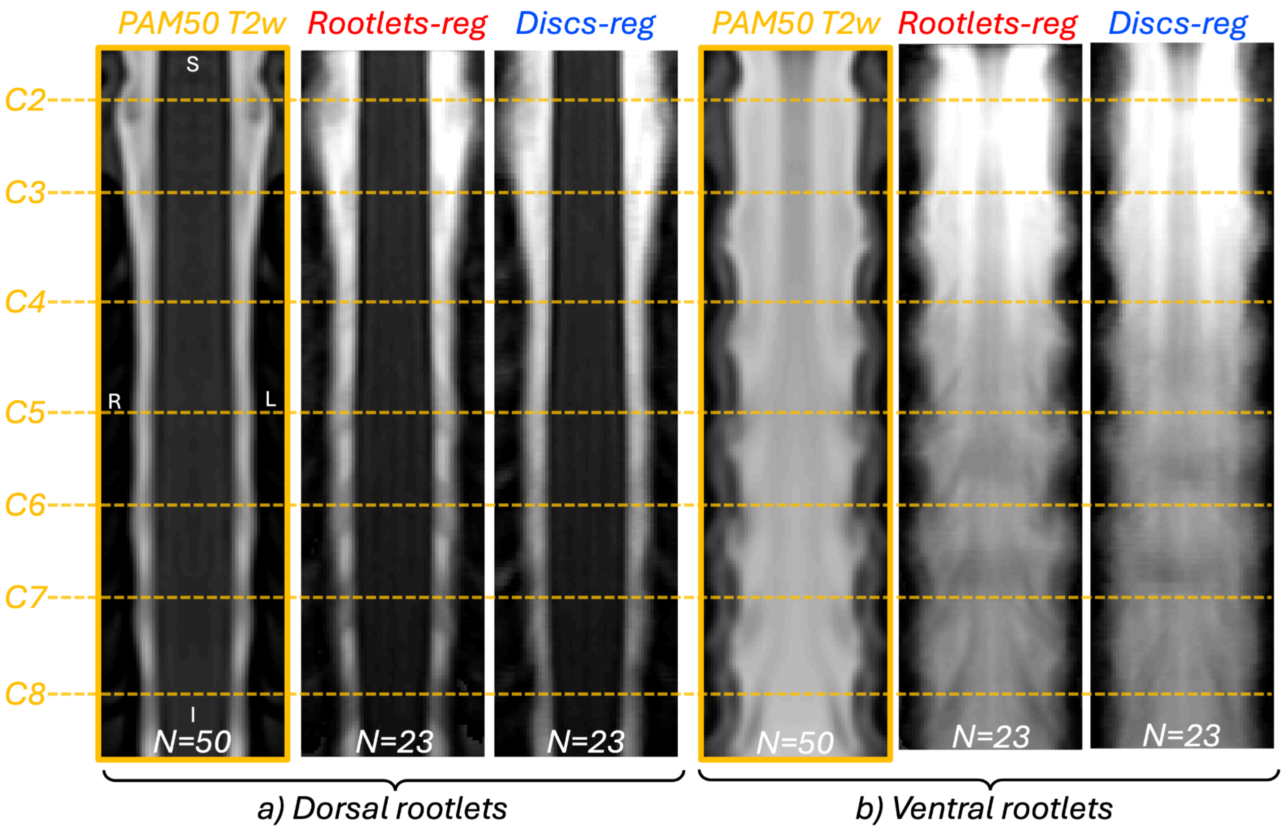

Figure S3. *Mean T2w image (n=23) registered to the template using rootlet-based (red) or disc-based (blue) registration. The PAM50 T2w image (n=50) is also shown (orange). The center of spinal levels C2 to C8 in the PAM50 template is marked by an orange dashed line.* ***a)*** *Example coronal slice (y=67) showing dorsal rootlets.* ***b)*** *Example coronal slice (y=78) showing ventral rootlets in PAM50 template space. Red arrows indicate an example of a good delineation of spinal rootlets.*
